# Supplementary figures and images for: Transmission and diversity of Schistosoma haematobium and S. bovis and their freshwater intermediate snail hosts Bulinus globosus and B. nasutus in the Zanzibar Archipelago, United Republic of Tanzania
Source: PLoS Negl Trop Dis. 2022 Jul 5;16(7):e0010585. doi: 10.1371/journal.pntd.0010585 (PMC9286283; doi:10.1371/journal.pntd.0010585)

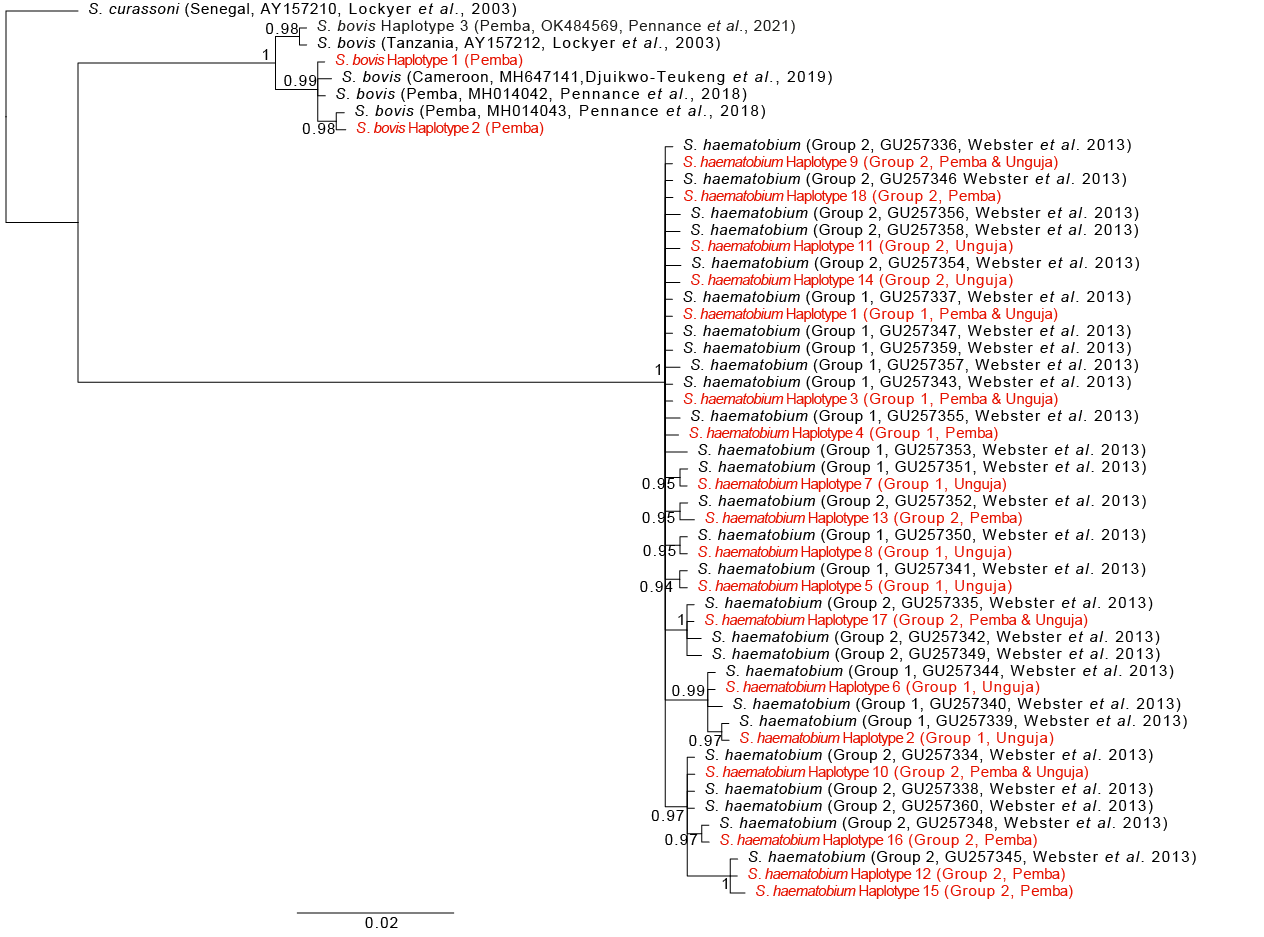

Supplement: S1 Fig — Phylogenetic tree constructed using Bayesian inference in MrBayes v3.2.7a [51] under the HKY + I model (-lnL = 1809.8890, AIC 3629.7781, ASDOSF < 0.01 at 1,791,000 generations). Branches with <0.95 posterior probability are collapsed. The branch length scale bar indicates the number of substitutions per site. Text in red indicates Schistosoma haplotypes generated in the current study. (TIF) [file pntd.0010585.s007.tif]
