# Supplementary material for: Transmission and diversity of Schistosoma haematobium and S. bovis and their freshwater intermediate snail hosts Bulinus globosus and B. nasutus in the Zanzibar Archipelago, United Republic of Tanzania
Source: PLoS Negl Trop Dis. 2022 Jul 5;16(7):e0010585. doi: 10.1371/journal.pntd.0010585 (PMC9286283; doi:10.1371/journal.pntd.0010585)
Supplement: Attachment — Submitted filename: PNTD-D-21-01740_Rebuttal_FINAL.docx [file pntd.0010585.s008.docx]

Dr. Stephen W. Attwood and Dr. Simone Haeberlein	London, 29^th^ April 2022

Associate and Deputy Editors, PLOS Neglected Tropical Diseases

**Submission of a new original research article to PLOS NTDs**

Dear Drs. Attwood and Haeberlein,

It is our pleasure to send this resubmission to you and your team at PLOS Neglected Tropical Diseases of manuscript entitled “**Transmission and diversity of *Schistosoma haematobium* and *S. bovis* and their freshwater intermediate snails hosts *Bulinus globosus* and *B. nasutus* in the Zanzibar Archipelago, United Republic of Tanzania**” (PNTD-D-21-01740). We thank the three reviewers for their interesting and useful comments, which we believe have further improved the quality of the manuscript for publication in PLOS NTDs. The comments and recommendations given by the reviewers have been accepted and incorporated into the manuscript, along with our rationale for the changes made in each instance.

In an effort to thoroughly and methodically respond to the reviewers comments, some of which overlap, we have split our response into 9 sections, including the reviewer comments highlighted in either blue (Reviewer #1), yellow (Reviewer #2) and green (Reviewer #3) relating to each topic and our responses to these in red. A final section of other corrections (including formatting and language corrections) that do not fit into these bigger topics are given at the end of this document following the same format.

We hope the reviewers and editors are more than happy with these amendments and responses and that the manuscript can move towards publication with this revised version.

Yours sincerely,

Drs. Tom Pennance and Bonnie Webster (on behalf of all authors)

Authors response to reviewer comments

**1) Snail identifications**

Reviewer #2: Overall, the methods employed seem reasonably sound. However I have some serious concerns regarding the number of snails and cercariae examined in the study.

My principal concern relates to the identification of the snails. Table 1 shows that a very large number of snails have been collected in the study but that only a very small number of these snails have been identified using molecular methods. As I initially read the methods section I assumed (as it turns out incorrectly) that all snails collected in the study had been identified (presumably using morphological methods) with molecular methods used to confirm identifications for just a small number of individuals. Such confirmation of identifications would have been perfectly acceptable but it turns out that the authors never actually bothered to identify the vast majority of snails they collected. Instead, we are told at the start of the results that the snails in a population were 'retrospectively' assigned as B. globosus or B nasutus based on the very limited number of samples for which molecular identifications were undertaken. So for the first site shown in Table 1 (Ukutine 21/2/2018) it is implied that 750 B. globosus snails were collected at that site, yet only 29 snails were actually identified as B. globosus. We see the same issue at all other sites. There would seem to be little point collecting such large numbers of snails from a site if you don't bother to look at them (to identify them) and it is most definitely not appropriate to 'retrospectively' identify snails in this way and in effect mislead the reader by implying that considerably more work has been undertaken than has actually been done.

The authors should therefore only present the data that they have. So for the site Ukutine in 21/2/2018, they collected 29 B. globosus specimens and B. globosus made up 100% of the snails identified at that site. Indeed this is exactly how most field studies are undertaken. We sample 20-30 individuals from a site which we identify and based on the proportions in our sample we determine the percentage of each species present. We don't imply that we have data for 750 snails when we do not.

Unfortunately for the authors, while sites like Ukutine with 29 identified specimens have been sampled reasonably well, other sites have not. Kizimbani 2016 has just 6 samples, Wawi 2016 has just 3 samples. The fact that you collected 199 snails from Kizimbani in 2016 and 149 snails from Wawi in 2016 is completely irrelevant as you didn't bother to look at them. Unfortunately there are many other sites with such pitiful numbers of identified snails.

The next question is does this matter i.e. is this likely to impact the results. The short answer is yes it does matter. Unfortunately, it is not the case that you only ever find monomorphic populations (with just a single species at a site) so sampling only a handful of individuals and inferring that this applies to the site as a whole is not enough. The authors own data in Table 1 shows that two sites have mixed populations of B. globosus and B. nasutus (Pujini 22/7/2018 and Chambani 24/10/2016). So for a site like Wawi with just 3 individuals identified and a site like Kizimbai with 6 individuals I have no confidence whatsoever that these populations are purely B. globosus and I wonder how many additional mixed populations might have been found if proper sample identification had been undertaken. I would suggest that a minimum of 20 individuals per site per time point would need to be identified for confidence.

Reviewer #2: My principal criticism of the results relates to the 'retrospective identification' of snail samples (or rather guessing the identification of the population at large based on a very small inadequate number of samples identified). Please see my comments above in the methods section of this review. I will not repeat these comments here but in essence the poor sampling undertaken (very few snails identified for some sites) renders all conclusions re presence or absence of B. globosus/ B. nasutus unsound.

Reviewer #2: Certainly some of the conclusions are not supported by the data presented. I have no confidence in conclusions relating to the presence/absence of B.globosus/B. nasutus at collection sites due to the inadequate number of snails identified at many of these sites.

However, I have confidence in the conclusion that B. nasutus acts as an intermediate host for S.haemotobium and is thus an important consideration in schistosomiasis control in humans.

Reviewer #2: There is some good data here and the finding that B.nasutus acts as an intermediate host of S. haematobium is of particular interest. Unfortunately, this very interesting finding is overshadowed by the inadequate number of snails identified at many of the collection sites. It is simply not safe to conclude that just a single species of snail is present at a site when only a handful of snails have been identified. The findings relating to the presence/absence of B.globosus/B.nasutus at sites on Zanzibar therefore cannot  be trusted. I therefore do not consider that the paper can be published in its present form.

However, I do note that the authors have undertaken excellent sample collections from Zanzibar with large numbers of snails collected from many sites. I would therefore encourage the authors to revisit their data and to undertake identifications of an adequate number of snails (minimum 20 per site per time point) and resubmit.

**Reviewer #3:** Second, the authors infer that all the snail individuals found in a given site belong to the species identified by sequencing 1-29 individuals. In my view, this is a very risky (and wrong) assumption and should be avoided. It is very likely that some of the sampled sites have more than one Bulinus species. In fact, the authors found two species in one of the sites. The authors should clarify the number of snail individuals that have been identified at the species level. They could say that XX individuals have been identify at the species level while XXX individuals remain only identified at the genus level. They could mention in the Discussion section that communities with more than one Bulinus species are common.

We thank the reviewer for this suggestion and have added in clarification that 10,606 snails remain only identified to the genus level, *Bulinus*, in the results section clearly reported.

**Reviewer #3:** Line 302: I think that this assumption is incorrect (see comment above):
“from that site were restrospectively assigned as B. globosus or B.
nasutus based on these identifications.”.

Line 326: Again I think that this assumption in not correct: “Where >1
snail was identified per site, each site was either inhabited solely by B.
globosus or B. nasutus”.

First of all, the authors would like to apologise if the reviewers felt misled whilst reading the sections on the number of snails identified through molecular methods, this was unintentional and we want to make as clear as possible to the readers.

As has been stated throughout the manuscript and emphasized more so now with relevant edits in the introduction and methods following the helpful comments by the reviewers, the identification of B. globosus and *B. nasutus* by shell morphology alone is not sufficient for accurately identifying the sample to the species. So although the collectors who are experienced malacologists did bother to examine each snail collected from the field to assure it was of the *Bulinus africans* species group, it is a largely futile effort to designate the species based on morphology alone. This is because far too much overlap in conchological features exists between these two species *B. globosus* and *B. nasutus* in Zanzibar, as has been demonstrated with publications referenced in the manuscript (i.e. Stothard et al. 1997). Therefore, the tried and tested method for species identification whilst providing insight on evolutionary relationships, which has been employed here, is *cox*1 sequencing. This form of molecular identification takes additional time for gDNA extraction, amplification, purification, sequencing and analysis as we are sure the reviewers are aware. During this study, we were restricted with both time and funding to complete this identification for all >11,000 snails, and therefore opted for a broad and systematic approach for sequencing a subset snails from each site and each survey (when multiple surveys conducted) as is outlined in the methods.

The reason why we initially retrospectively assigned snails to each species from a small number of identifications, was because all previous findings and evidence points at a clearly allopatric distribution of the species on both islands. Our finding that *B. globosus* and *B. nasutus* at the same site is the first to demonstrate cohabitation in Zanzibar. We have therefore withdrawn comment and referral to the ‘retrospective identification’ of snails, as we agree with the reviewers that this is not appropriate, especially when we have observed overlap in our results. It was rather unnecessary considering the aims of this study. We therefore refer to the 10,606 snails that molecular identification was not performed as *Bulinus africanus* group species of the *Bulinus* genus in the revised manuscript.

The authors are well aware of the need for better *Bulinus* species diagnostics, in papers such as ours. In fact, we are developing a rapid diagnostic marker to differentiate *B. globosus* and *B. nasutus*. However, this tool is not yet at the capacity to run the assay on all snails, and it also requires some further validation before employing on a large sample set such as this, as well as the funding to do so.

We would like to thank the reviewers for their comments that have helped us change the language in the methods and results to be clear that only a subsets of snails, the maximum that was possible in the scope of this study, were identified, and indeed this subset cannot be representative of all snails at the site. We have therefore changed the language regarding this in all the relevant parts of the manuscript, including as a limitation to the study in the discussion section.

**2) Cercariae identifications and snail infection identifications**

Reviewer #2: As well as my concerns re snail identifications, I also have some concerns over the number of cercariae samples. Rather than tell us in the methods that a subset were sampled please be clear and tell us exactly how many cercariae were sampled.

Reviewer #3: Line 247: Again, the total number of individuals analyzed is not clear. It
is very difficult to infer how many cercariae per snail have been
analyzed? DNA was extracted individually? Or DNA from cercariae
belonging to one infected snail were collectively extracted?

The number of cercariae samples processed for molecular identification is clearly stated in the methods, and all resulting information is in supplementary table 4 which provides exact detail of number of cercariae identified from each snail, and the haplotypes of these:

‘From each infected snail either two or six S*chistosoma* cercariae were processed individually for molecular identification. Six cercariae were individually processed for each infected snail collected between 2016 and 2019 (n=20 snails) and two cercariae for snails collected as part of the ZEST study (n=69 snails) [16–18] and made available via SCAN (Table S4).’

Reviewer #2: I do also wonder why the authors have used cercarial shedding to determine infection rate rather than use PCR based methods of infection detection. Such PCR based methods are much simpler and avoid inaccuracy due to missed pre-patent individuals (though having said this the authors do make an effort to screen for cercaria on more than one occasion; at least for the majority of populations).

PCR based detection of schistosome infection (molecular xenomonitoring) of snails from Pemba was performed as part of another unpublished study and therefore is not relevant for this current paper dealing with the distribution and diversity of *Bulinus* and *Schistosoma* species transmission across both Pemba and Unguja. We have however added a sentence to the study limitations section in the discussion detailing that in a future study, PCR based methods would be of interest to use:

“In a future study, it would also be of interest to use PCR based methods to identify pre-patent S. haematobium and S. bovis infections in snails to further assess the total number of snails that have been exposed to schistosomes but are not currently contributing to transmission [77].”

As stated in the introduction, our main aim of this study was to determine those snail species involved in transmission of schistosome species, it is well documented that finding a pre-patent infection does not mean that that snail is compatible and contributes to transmission.

**3) Use of term paraphyletic**

Reviewer #1: Line 336. Paraphyletic with respect to what? In each case, they look like sister clades to me. Please check that the rest of this description of the tree in Fig. 3 is accurate.

Reviewer #2: Paraphyletic would seem inappropriate when referring to the clades in the tree. Remove and just refer to them as clades.

Line 459. Two distinct clades – I agree. But each of these seems to consist of two distinct subclades.  Might that have some significance?

Corrected, removed reference to paraphyletic and instead described correctly as two clades with sister groups within.

**4) Table 1**

Reviewer #2: Please also note that the results text does not seem to match the data in Table 1. In the text we are told that just 1 site has a mixed population of B. globosus and B. nasutus. In Table 1, two sites are shown to have a mixed population of B. globosus and B. nasutus. I would also like to see some number in Table 1 for these 2 sites. How many of the individuals identified were B. globosus and how many B. nasutus.

Reviewer #3: Table 1: this table is very extensive for the main text. I would include it
in Supp. Mat. In the main text, the authors should include, however, an
abstract of this table mention the total number of sampled snails, the total
number analyzed from the museum collection, the total number of
molecularly identified snails, etc. It is very difficult to find in the
manuscript this information? How many snails have been collected?
How many snails have molecularly analyzed?

Thanks to the reviewers for looking over, however the Table 1 does not show the number of Bulinus by site, but by shehia (which sites are nested in as described in the methods and as is shown in the table column headers). Therefore, the column representing *Bulinus spp*., which the reviewer is referring to, is showing the result for all sites within that shehia and in Chambani, there are sites that have B. globosus, and other sites that have B. nasutus as identified through the subset of snails molecularly identified (and displayed and discussed in the results and Fig 2). 112 sites were surveyed between 2016 and 2019, some more than once, hence this is a much larger dataset that has been summarized to show the collections by shehia for the reader to interpret.

Although the Table is expansive, we believe it to be absolutely necessary for the reader to interpret the collection effort over several years and time points and regions in an effort to collect infected *Bulinus* snails, and also to molecularly identify snails representing each shehia surveyed at each time point. A shortened version of this table as suggested by Reviewer #3 would be a repeat of what is given in the results / methods section.

**5) How schistosome species present on Zanzibar are reported**

Reviewer #2: It is stated in the manuscript that Schistosomiasis transmission on Zanzibar was believed to involve a single schistosome species (Schistosoma haematobium) transmitted via a single intermediate host species (Bulinus globosus). Statements to this effect are not accurate and should be removed from the manuscript. I do appreciate that the authors are keen to enhance the impact of their work in this paper but S.bovis has previously been reported on Zanzibar by the authors of this paper in a previous paper from 2018. The discovery of S. bovis on Zanzibar is thus not a discovery new to this paper and should not be presented as such.

Thanks to the reviewer #2 for highlighting this. The section in the introduction that is being referred to by the reviewer has been edited so that it is clear that both species of schistosome were known to be present in Zanzibar before the current study findings were reported. It is not presented as a new finding in the results manuscript, but presented as confirming previous finding that this schistosome species is focally established on Pemba.

**6) Number of infected snails**

My main point of confusion concerns the number of infected snails. In the abstract and in line 364, 89 snails were said to be infected. This probably matches the number in Table S2, but I haven’t checked in detail. In other places in the paper, it is stated that 20 snails were infected (line 307).  In Table 1 and Table S1, I can identify 22 infected snails. In Table 5, there seem to be 21 snails. I assume that the 69 unidentified snails are those referred to in line 251. These numbers need to be clarified.

In the methods, it is clear that 20 snails were identified during 2016 and 2019 to be infected and cercariae collected, and that also cercariae that were collected from 69 other snails during the ZEST study, were identified. The reason why 22 infected snails are listed in Table 1 and Table S1 is because this Table represents the number of snails (not cercariae collected from snails) identified, 20 of which are from Pemba between 2016 and 2019 and two snails identified from the SCAN repository that were identified as infected with *S. haematobium* previously and included to molecularly identify the snails.

We have made this clearer now also in the results section for the reader also:

‘From the subset of cercariae identified from 89 of the infected Bulinus collected from Zanzibar between 2016 and 2019 (n=20) and identified during the ZEST study (n=69), 82 were shedding S. haematobium with 18 different haplotypes and the remaining seven snails were shedding S. bovis cercariae of two haplotypes as presented in Table S3 and Table S4.’

**7) Species of schistosome present on Zanzibar**

**Reviewer #3:** There are also some confusing sentences or incorrect assumptions. For
example, I think that these facts should not be related (see below): “Although S.
haematobium on Zanzibar is genetically more diverse in comparison to mainland
Africa, with the presence of both Group 1 (mainland Africa) and the more diverse
Group 2 (Indian Ocean Islands) strains, it remained the only Schistosoma species
identified on the islands”.
**Thanks to the reviewer for picking this up. This passage has now been edited to avoid the confusion of linking *S. haematobium* diversity with the presence of only a single species which was not our intention. The sentence now reads:**

**‘***Schistosoma haematobium* populations on Zanzibar are considered more genetically diverse in comparison to mainland African populations, with the presence of both Group 1 (mainland Africa) and the more diverse Group 2 (Indian Ocean Islands) strains [27,28].’

**8) Role of *Bulinus nasutus* as an intermediate host**

**Reviewer #3:** There are two other major issues that should be emended. First, the authors say the manuscript demonstrates that the snail species B. nasutus could play a minor role in transmitting Schistosoma in Zanzibar because they found one individual infected with the parasite. However, I think that the fact that suggest that this snail species could be playing a role in transmitting the parasite is not having found one infected snail but, instead, the fact that this snail species transmits the disease in Kenya and Tanzania. There is no reason to believe, I think, that if the snail species is present in the archipelago, it would not transmit the disease. This fact should clarify because it is very confusing as it is.

**We made this clearer in the manuscript introduction, and more so in the discussion that there was good evidence that *B. nasutus* was completely refractory to *S. haematobium* infection on Zanzibar. Zanzibar has been a completely different transmission system from Kenya and Tanzania, where the author is correct, this species is involved in transmission. This shows the complexity of compatibility between snails and schistosomes, and is what we were trying to highlight in our paper regarding mainland type schistosome parasites being transmitted by *B. nasutus*. It could be hypothesised that previous experimental infections with *B. nasutus*, before we had an understanding of the *S. haematobium* diversity that may effect resistance / susceptibility of the snail (allele matching model), only used more abundant Indian Ocean strains of *S. haematobium*, and hence leading to a resistant combination. This kind of resistance is well documented in the snail / schistosome literature.**

**9) Reduction and changes of Figures and Tables**

Reviewer #3: I found the manuscript very interesting for those with closely related research interests: parasitologists studying zoonotic pathogens in Tanzania and other Africa countries, for instance. The methodology is appropriate. I am not a specialist in schistosomiasis nor in Bulinus snails but I think that references are fine. I found one major issue, however, that should be ameliorated to make the manuscript more attractive and easier to follow by the reader. Results should be shorten and presented more straightforward to make the manuscript clearer. This issue can be easily achieved with some rewriting. The way the authors present the results (text, figures and tables) is confusing or too complicated (see below). I would reduce the number of figures and tables. I would delete some tables and figures and modify others (see below). I am not sure if the journal allow supplementary material. If it does, I would move some figures to supplementary material (see comment below).

**Reviewer #3:** Figure 1: I think this figure is relevant but I would midify it and include it in the Result section. A figure that compares previous findings by Stothard et al. [23], Pennance et al. [28,30] and Knopp et al. [18] with the results from this manuscript would be more useful. The reader would better visualize which are the inputs of this manuscript. The authors could highlight the regions where the snail and parasite species have been found and put dots in the localities from this study where the authors have found snail and parasite. The manuscript would be become clearer and the figure would be more helpful to the reader.

o Figure 2: I would combine Figures 1 and 2. Se comment above.

We thank the reviewer for this recommendation. In fact, this suggestion was also what was originally considered when planning the figures for the manuscript, but we decided to keep the figures 1 and 2 separate for this final version for two reasons (and after careful consideration we believe they should remain like this.) The first reason for this is that Figure 1 provides the background for the current studies relevance with an overview to the reader of our current knowledge of *Schistosoma haematobium* endemicity across the Zanzibar islands, and for the first time combines this with the current findings on *Bulinus* distribution across the islands. This is the first time that the most recent epidemiological data (from ZEST studies) has been mapped with *Bulinus* distribution, and a key feature highlighted is the overlap between *S. haematobium* infections and *B. nasutus* distribution on the east of Pemba Island. This demonstrated the need for us to look further into the *Bulinus* spp. distribution on Pemba in particular, and assess the *Schistosoma* infections from these snails. Figure 2 displays the results of the current study, which for the most part agree with what was displayed in Figure 1, however shows that there is some overlap between *B. globosus* and *B. nasutus* to the east of the island, which was previously thought (as shown in Figure 1) to be separated. This is a key finding of our study as discussed in the manuscript, and therefore necessitates both figures 1 and 2.

The second, and maybe more important reason that both figure 1 and 2 are not combined, is that the resulting combined Figure is very difficult to interpret because of there being so much information presented, especially with the overlap of colours showing different *Bulinus* species distributions (both from previous studies and current study), the shehias surveyed, the endemicity of *Schistosoma haematobium* etc. A conjoined figure would not be easily interpreted by the reader, and be less useful overall.

We hope the editors and reviewers agree with this sentiment and that the figures can be kept separate. If the editors or reviewers deem necessary, one of the figures (Figure 1) could be moved to the supplementary material, however we feel this would take away significantly from the manuscript.

Finally, following the recommendations for reducing the Tables and Figures in the results, please see response to further comments below.

o I would move Tables 2, 3, 4 and 5 to Supp. Mat. I would present instead a single table that resume the results of the manuscript. These tables are very descriptive as they are but I think the reader would need some table that resume and illustrate the results of the manuscript.

**Thanks for this suggestion, the four tables (Table 2 – 5) have been moved to the supplementary material**

o I would move Figures 3 and 5 to Supp. Mat.

Figure 5 has been moved to the supplementary material, as we agree that this figure is not necessary in the main text considering that a haplotype tree for the *Schistosoma* species is also shown. However, the authors would like Figure 3 to remain in the text as this is an important figure displaying the *cox*1 diversity of *B. globosus* and *B. nasutus* on Zanzibar, which has not been presented in such a detailed fashion before.

This version of the manuscript has 5 figures and 5 tables. I think it too much. I
think that two figures (one showing a map with previous and current results and
other showing the parasite haplotypes) and a table resuming the manuscript
results are more than enough for such a manuscript.

We have reduced the number of Tables down from five to one, and have also removed one of the figures. As previously mentioned, the other four figures we believe are important outputs from the current study, or provide necessary background to the current study (i.e. Fig 1), and see no reason why these cannot remain in the published manuscript to provide the reader with maximum amount of information on this subject.

**Line by Line comments**

**Reviewer #1:**

Line 33. “… allopatric for S. haematobium…” What does that mean?

In biology, allopatric refers to the presence of a single, in this case species, non-overlapping distribution of an organism. We use the terminology here to highlight that only *S. haematobium* was known to be present across Zanzibar, until the recent identification of S. bovis, which the overlaps in distribution (i.e. now sympatric). We have now included ‘sympatric’ in this sentence, to highlight this change to aid in future interpretation:

‘Additionally, the Archipelago previously considered to be allopatric for *S. haematobium* has recently reported sympatric transmission of *S. bovis*, a parasite of ruminants’

Line 48 and elsewhere. Which islands are regarded as being members of the Indian Ocean Islands?

There are many islands in the Indian Ocean, and too many to list here. We use the terminology here to match that in a previous PLOS NTDs paper (Webster et al. 2006, Genetic Diversity within Schistosoma haematobium: DNA Barcoding Reveals Two Distinct Groups) that identified the two distinct groups of S. haematobium, referred to as mainland and Indian Ocean type.

Lines 81-83. Low-level transmission maintained through exposure to cercariae released from infected snails.  This seems to be stating the obvious. What point is being made?

Upon rereading, the point seems clear to the authors, that although humans are being treated, it is the reservoir of infection in snails (i.e. highlighting the importance of snail surveillance and control relevant to the paper) that is reinfecting populations and causing bounce back of prevalence after treating.

Line 171 and many other places. I would not personally use “represents”. Maybe “indicates”?

Thanks for the suggestion, this has been changed throughout.

Lines 181-182. “…searching as much of the perimeter of the water body as possible…”?

We are not sure on the reviewers exact issue with this sentence, however we edited to say ‘accessible perimeter’ if that helps clarify that waterbody edges were searched (where snails reside) where they could be accessed. Some deep water or steep banks restrict access for collectors to those parts of the waterbody. Sentence now reads:

‘Each site was surveyed for 15 minutes by three collectors, starting from access points and then searching as much of the accessible perimeter of the waterbody during this time.’

Lines 207-209. This sentence should be rephrased or split.

Corrected. Sentences now split:

‘Snails were fed on dried lettuce when all lettuce in the tank had been eaten. Dead snails were removed from the aquaria daily.’

Lines 238-241. As far as I can tell, you sequenced DNA from 510 snails, not the 1009 snails as implied here. Maybe clarify that here

Sorry for the misinterpretation, we have added a comma to the sentence to try and make clear that 510 is the total, and that this includes 11 snails from Unguja and the 499 from Pemba as stated:

‘DNA was extracted from a total of 510 Bulinus spp. snails, from Unguja (11 snails collected from 8 sites) and Pemba (499 snails collected from 63 sites).’

Line 248. The cercariae were apparently processed individually, not pooled. Perhaps make that clear.

Corrected:

‘From each infected snail either two or six *Schistosoma* cercariae were processed individually for molecular identification’

Lines 324, 418 and maybe elsewhere.  Don’t start a sentence with an abbreviated genus name.

Thanks for catching this, all instances corrected where genus at the start of a sentence.

Line 368. “co-infections” here, with a hyphen. Without a hyphen elsewhere.

Changed to ‘coinfection’

Fig. 2. At least in the review pdf, the symbols on figures are rather small and hard to read. The coloured circles in Fig. 2 could be made larger for a start.  In Fig. 4, the lettering is too small.

The circles on Figure 2 are restricted in size because of overlap and the scale of the map to represent all the data. The high quality figure is included for the final manuscript. As for Figure 4 too, we hope this to be displayed in a higher quality format in the final manuscript (as was uploaded separately to the Figure in the text). The text is appropriately sized for this figure that make it easiest to interpret. High quality versions of these figures are uploaded with the manuscript.

Is Fig 5 necessary, given Fig. 4? Also, inclusion of both species separated by one very long branch has the effect of rendering the branches within S. haematobium (in particular) too short to be visualised properly. Indeed, Group 1 and Group 2 haplotypes seem to be thoroughly intermingled in this tree.

Figure 5 has now been moved to the supplementary material (see above, now Fig S1). As for the second point on branch length, this is just an outcome of the phylogenetic analysis and cannot be changed. The authors believe this can remain as is and it would not provide anything beneficial to plot both species separately and would necessitate additional figures of which is trying to be reduced.

Line 494. Local strain of…

Corrected

Reviewer #3:

o It should better specify the geographic area studied. Include “Zanzibar Archipelago, Tanzania” in the title.

The title has been amended to “Transmission and diversity of *Schistosoma haematobium* and *S. bovis* and their freshwater intermediate snail hosts *Bulinus globosus* and *B. nasutus* in the Zanzibar Archipelago, United Republic of Tanzania”

o Delete the following phrase “The involvement of B. nasutus (a snail species that occupies geographically distinct regions on the Archipelago) in S. haematobium transmission has previously been debated.”. It is the second phrase in the abstract and is very confusing. It does not saying anything as it is.

The sentence has now been edited to a more direct point stating that the role of *B. nasutus* is unclear. “Following multiple studies, it has remained unclear if *B. nasutus* (a snail species that occupies geographically distinct regions on the Archipelago) is involved in *S. haematobium* transmission on Zanzibar.”

o Line 52:host or parasite intraspecific variability?

*Schistosoma haematobium* intraspecific variability, now changed to: “We provide preliminary evidence that intraspecific variability of *S. haematobium* may increase the transmission potential of *S. haematobium* due to the expanded intermediate host range, and that the presence of *S. bovis* complicates the environmental surveillance of schistosome infections.”

o Line 53: “intraspecific variability may increase the transmission potential of S. haeamtobium due to the expanded intermediate host range”. I think that both facts are not necessarily linked.

Line 52. “…evidence that intraspecific variability may increase the transmission potential…”. Th evidence is not very convincing to me.

As demonstrated in the paper, we are highlighting here the fact that mainland group *S. haematobium* cercariae, that are transmitted by *B. nasutus* on mainland Africa have been identified here in Pemba being shed from a *B. nasutus* snail. As all previous evidence, infection challenges and malacological surveys have not identified shedding *B. nasutus*, we hypothesize that the new shedding from *B. nasutus* could be due to allele matching, as has been shown in the manuscript supported by previous literature on compatibility between geographical strains of *S. haematobium* and *Bulinus* species. We also state this to be preliminary evidence throughout, and are clearly hypothesizing as a means for spurring future investigation. We have edited this sentence to make more relevant to the current localized findings on Pemba:

‘We provide preliminary evidence that intraspecific variability *of S. haematobium* on Pemba may increase the transmission potential of *S. haematobium* locally due to the expanded intermediate host range.’

o The total number of collected snails and the number of sampled sites should be present in the abstracts since it is one of the highlights of the manuscript. There is a great sample effort and that should be emphasized.

This has now been added to the remodeled abstract, giving more detail to the number of samples collected from new collections, those incorporated from archived collections, the number of snails and cercariae identified.

Introduction:
o Line 105: Snail habitat preferences?

See the reference provided, the Stothard (2000) study shows the differences in distribution of *Bulinus* on Zanzibar based on water hardness. I have now added to sentence to make clear that this is what is being referred to:

“*Schistosoma haematobium* transmission in the past couple of decades was therefore considered to be restricted across the islands to only freshwater bodies where *B. globosus* was present (Fig 1), with the distribution of *B. globosus* on the islands also being constrained by this species habitat preferences such as water hardness [23].”

o Line 106-109: “Although S. haematobium on Zanzibar is genetically more diverse in comparison to mainland Africa, with the presence of both Group 1 (mainland Africa) and the more diverse Group 2 (Indian Ocean Islands) strains, it remained the only Schistosoma species identified on the islands”. I think that both facts are independent. In fact, the authors say latter that this is not true: S. haematobium is genetically diverse in the islands and it is not the only Schistosoma species present in this area.

Thank you to the reviewer for highlighting this. This section has now been completely reworked and avoids confusion that *S. haematobium* diversity is a pre-determinate for it being the only *Schistosoma* species present on the island, and it also states clearly that *S. bovis* is also present:

“*Schistosoma haematobium* populations on Zanzibar are considered more genetically diverse in comparison to mainland African populations, with the presence of both Group 1 (mainland Africa) and the more diverse Group 2 (Indian Ocean Islands) strains [27,28]. However, it was not until recently that a second *Schistosoma* species, *S. bovis* which infects ruminants, was identified as being transmitted by *B. globosus* on Pemba [29]. Surveillance of *Schistosoma* transmission in Zanzibar is therefore complicated by not only the presence of two morphologically indistinguishable larval Schistosoma species shed from Bulinus snails, but also two morphologically overlapping Bulinus species.”

o Line 178: “snails were identified using shell morphology to their genera”. The authors should clarify that species cannot be morphologically identified at species level. And that thus why molecular analysis are needed.

We believe the reviewer is referring to the identification of *Bulinus* to the species level, but we are referring to all snails observed at the site (i.e. information on other snail genera, Pila, Lanistes, Cleopatra etc were also collected). Also, we made clear that *B. forskalii* group snails were not collected. To try and make this more apparent, we have changed the sentence to:

“At each site, snails were identified using shell morphology to their genera, with any non *Bulinus africanus* group snails (namely *B. forskalii* group snails) being returned to the collection site.”

o Line 205: “after being stored for at least 48 hours in transparent 15L water containers (allowing for any sediment to settle and eliminate the risk of introducing live schistosome eggs, miracidia or cercariae into the aquaria)”. I am not an expert in Schistosoma. Cannot eggs live more than 48H?

Once in freshwater, eggs should hatch within a few hours and then once hatched are not alive for more than 24 hours. 48 hours is therefore ample time to avoid miracidia contamination

o Line 242: “before being collapsed into cox1 haplotype groups.” Which software was used for identifying haplotypes?

Sequencher v5.4.6 – this programme is stated in the methods but may have been missed by the reviewer.

o Line 301: “a total of 11,110 B. globosus and B. nasutus were collected from ...” What about the third undescribed species?

We believe this comment to be a misunderstanding as there is not a third undescribed species that was collected. The reviewer may be referring to the undescribed *B. forskalii* group species mentioned in the abstract, and we have therefore modified an earlier sentence (see above) to drive home that these were not sampled since not of importance for *S. haematobium* group species transmission in East Africa.

o Line 324. Replace “B. nasutus” by “Bulinus nasutus”.

Caption of Figure 2 changed

o Line 366: Replace “S2 Table” by “Table S2”.

Replaced all supplementary table referrals to Table S2, Table S1 etc.

o Figure 4 is very informative. I really like it.

Thank you to the reviewer for good feedback.

Discussion
o Replace 0.18% by 0.2%. Decimals in prevalence should be only used when prevalence is lower than 1% and higher than 99%. See

<https://nam12.safelinks.protection.outlook.com/?url=http%3A%2F%2Fadc.bmj.com%2Fcontent%2F100%2F7%2F608&amp;data=04%7C01%7Ctpennance%40westernu.edu%7C5676f59cb160436aeee308d9ff7f8d8f%7C25a6f4f8d912492a84df376754ba693e%7C0%7C0%7C637821745580935752%7CUnknown%7CTWFpbGZsb3d8eyJWIjoiMC4wLjAwMDAiLCJQIjoiV2luMzIiLCJBTiI6Ik1haWwiLCJXVCI6Mn0%3D%7C3000&amp;sdata=jgAbH9YFixBj%2FGouBB9m2siH7T0ib5ZOvpAze%2FLNobs%3D&amp;reserved=0>
Corrected, thank you for the information.

o Please, explain the following idea mentioned in the Abstract: “preliminary evidence that intraspecific variability may increase the transmission potential of S. haematobium”. I found it relevant and it should be better explained in the Discussion section. Why the authors believe that intraspecific variability may increase the transmission of a disease?

This is discussed in a large paragraph present in the discussion, under the section ‘A ‘new’ intermediate host of *Schistosoma haematobium* on Pemba: *Bulinus nasutus’*, that deals with what we know already on snail/schistosome compatibility, strain dependent compatibility between geographical isolates etc. The discussion section, which has been edited to make sure this point comes across more so, is as follows:

“*Schistosoma haematobium* cercariae shed from the intermediate snail hosts, analysed here, fall into the two known *S. haematobium* haplotype groups (1 and 2) previously identified from miracidia collected from infected humans [27,28]. The cox1 haplotypes of cercariae shed from *B. nasutus* were identified as Group 1, a group predominantly associated with African mainland *S. haematobium* populations. Possibly only Group 1 S. haematobium is compatible with Pemba *B. nasutus*, since this same snail species acts as an intermediate host in East Africa [31–37], however more samples would be needed to test this hypothesis. *Schistosoma* strain specific interactions/compatibilities with intermediate host snails based on differential immune responses, have been well studied and established in strains of *S. mansoni* and *B. glabrata* (as summarised in [67]). However, relatively little is understood regarding co-evolution of host/parasite compatibility between *S. haematobium* group strains and *Bulinus* species, save some studies investigating geographical isolates (see [68–72]). Such strain dependency or local adaptation reflects the patchy compatibility of *Bulinus* snail hosts of *Schistosoma* generally [73], discussed in a previous study in Tanzania, where experimental infections of *B. nasutus* using a local strain *S. haematobium* that usually infects *B. globosus* were unsuccessful [74]. As demonstrated again here, *B. globosus* remains the primary host of S. haematobium on Zanzibar, but endemic *B. nasutus* may also play a minor role in transmission involving specific *S. haematobium* strains, complicating future monitoring. This hypothesis also provides some explanation to how ‘intermittent’ and/or ‘unstable’ transmission was historically maintained in areas of Zanzibar (such as south Unguja) where only *B. nasutus* is present currently [7,9,10,75,76].”

Avoid using symbols (*, a , b ...) in the caption section. It is confusing and it takes time to understand what the authors meant. Try to include these remarks in the caption. For instance, in Figure 1, the sentences “* Bulinus spp. distribution inferred from Stothard et al. [23] and Pennance et al. [28,30].” and “φ Schistosoma haematobium infection distribution interpreted from Knopp et al. [18].” could directly appear in the caption without including the symbols.

This has been corrected, Fig. 1 has been adjusted to not include the symbols and the caption has been edited.
