# Supplementary material for: Transmission and diversity of Schistosoma haematobium and S. bovis and their freshwater intermediate snail hosts Bulinus globosus and B. nasutus in the Zanzibar Archipelago, United Republic of Tanzania
Source: PLoS Negl Trop Dis. 2022 Jul 5;16(7):e0010585. doi: 10.1371/journal.pntd.0010585 (PMC9286283; doi:10.1371/journal.pntd.0010585)
Supplement: Attachment — Submitted filename: PNTD-D-21-01740_Rebuttal_2.docx [file pntd.0010585.s009.docx]

Dr. Stephen W. Attwood and Dr. Simone Haeberlein	London, 6^th^ June 2022

Associate and Deputy Editors, PLOS Neglected Tropical Diseases

**Submission of a new original research article to PLOS NTDs**

Dear Drs. Attwood and Haeberlein,

It is our pleasure to send this second revision to you and your team at PLOS Neglected Tropical Diseases of manuscript entitled “**Transmission and diversity of *Schistosoma haematobium* and *S. bovis* and their freshwater intermediate snails hosts *Bulinus globosus* and *B. nasutus* in the Zanzibar Archipelago, United Republic of Tanzania**” (PNTD-D-21-01740). We thank the three reviewers for their interesting and useful comments, on the original submitted of this manuscript, and the further comments provided by two reviewers on the revised version. Both stages of the review have further improved the quality of the manuscript for publication in PLOS NTDs. The minor comments and recommendations given by one reviewer to the first revised version have been accepted and incorporated into the further revised manuscript. Response to these are given bellow, with reviewer comments in black text and our responses to these in red.

We hope the reviewers and editors are more than happy with these amendments and responses and that the manuscript can move towards publication with this further revised version.

Yours sincerely,

Drs. Tom Pennance and Bonnie Webster (on behalf of all authors)

Authors response to reviewer comments

Reviewer #1: The paper is getting closer to being acceptable and will be a useful addition to the literature. I have few quibbles with the data but would like to appeal for greater clarity in the writing.

Thank you to the reviewer for taking the time to take another detailed look at the manuscript.

Line 33. My original question about “allopatric” still stands. In my opinion, the term is simply mis-used by the authors. Surely they mean that until recently S. haematobium was thought to be the only Schistosoma species on the archipelago, but that transmission of S. bovis has now been demonstrated there.

Thank you for the reviewer for reiterating this point. We have now changed the sentence to remove mention of the word allopatric:

“Additionally, *S. haematobium* was thought to be the only *Schistosoma* species present on the Zanzibar Archipelago until the sympatric transmission of *S. bovis*, a parasite of ruminants, was recently identified.”

Line 52. Again, Indian-Ocean Islands. I agree that there are many of these, but presumably the authors know which ones they mean. Are these the Zanzibar Archipelago and Mafia? Or does that number include Mauritius and Madagascar? Anywhere else? Clarity please.

Thank you for this comment. We have now added the specifics islands we are referring to (which are also listed in the puclication that is later referenced in the main body of text (Webster *et al*., 2012):

“By analysing a partial mitochondrial *cox*1 region and the nuclear ITS (1-5.8S-2) rDNA region of *Schistosoma* cercariae, we identified 18 *S. haematobium* and three *S. bovis* haplotypes representing populations associated with mainland Africa and the Indian Ocean Islands (Zanzibar, Madagascar, Mauritius and Mafia).”

Lines 88-89. Just say that low-level transmission is continuing. Many more words than required are used here.

We have reduced this sentence considerably, however still want to include that the reservoir of infection left over in snails is responsible for the continuation of schistosome transmission to tie into the rest of this paragraph. Sentence now reads:

“As prevalence of the infection in humans moves towards elimination in parts of sub-Saharan Africa following large-scale multi-year treatment programmes, it is apparent that low-level transmission is continuing, facilitated by the presence of freshwater snail intermediate hosts enabling reinfection [5,6].”

Line 116. Maybe an apostrophe after “species”?

Corrected.

Line 117. Clumsy phrasing “freshwater waterbodies”. Maybe “freshwater habitats” or something else.

Corrected to habitats, thanks for noticing.

Line 119. “overlapping with B. globosus”.

Corrected.

Line 149. “…between 2011 and 2019”. But 2016 is the year given elsewhere.

Thanks for the reviewer for catching this. We decided to be more clear and state the range of years that samples included in this study were collected (between 2005 and 2019):

“In this study we aimed to investigate the current transmission status of urogenital and bovine schistosomiasis on Pemba and Unguja islands by monitoring the species distributions and genetic diversity of *B. globosus* and *B. nasutus* and their associated *Schistosoma* spp. collected between 2005 and 2019.”

Sentence starting on line 320. Rephrase to remove the repetition of “remain(ing)”.

Thanks for noticing this. Now changed to other:

“The other 10,606 *Bulinus* collected remain only morphologically identified as being within the *Bulinus africanus* species group (*Bulinus* genus), since morphological differentiation between *B. globosus* and *B. nasutus* is not possible.”

Line 343. Are you implying that, where >1 snail was identified at a site, all other snails from that site were regarded as belonging to that species? The phrasing is poor.

We have improved the phrasing of this sentence, and with referral to the Table S1 as given in the text it is clear as to what is being referred to. We are only referring to those snails molecularly identified:

“Where >1 snail was molecularly identified per site, the snails were identified as either *B. globosus* or *B. nasutus* coming from the same site, except for from one waterbody on Pemba (Puj11, Fig 2B), where both species co-occurred in the same seasonal pond (Table S1).”

Line 347. This seems to be a place where “cohabiting” could be replaced with “sympatric”.

Removed cohabiting and did not replace with symnpatric since already stating that populations could be ‘mixed’. Sentence now reads:

“Based on the subset of molecularly identified *Bulinus*, no other mixed *B. globosus* and *B. nasutus* populations were observed, however this would have to be fully confirmed with further genetic analysis of a large set of snails from each water body.”

Lines 379-382. This sentence should be split and rephrased for clarity. As it stands, the subject of the sentence seems to be cercariae, but the authors are then clearly referring to snails.

This is a long, but necessary sentence especially after the last round of revision that then required more clarity on the origins of the snails in this section. We are referring to cercariae haplotypes identified from the infected snails. We have split the sentence with a comma and reduced some wording, and we believe the sentence to be clear regarding the number of haplotypes of *S. haematobium* and *S. bovis* identified form how many snails. Sentence now reads:

“From the subset of cercariae identified from 89 of the infected *Bulinus* collected from Zanzibar between 2016 and 2019 (n=20) and identified during the ZEST study (n=69), 82 were shedding *S. haematobium* with 18 different haplotypes and seven were shedding *S. bovis* with two haplotypes (Table S3 and Table S4).”

Lines 398-399. Maybe “…variants other than those previously sequenced...“

Sentence reduced and simplified and final sentence refined to only refer to ITS profiles as *cox*1 haplotypes already discussed:

“Comparison of mitochondrial *cox*1 and nuclear ITS profiles from *S. haematobium* cercariae showed no evidence of hybridisation between *S. haematobium* and *S. bovis*. No intraspecies variation in the ITS profiles were observed, with 100% match to reference data [27].

Line 428. Where are the risk maps?

Reference to risk maps, since misleading, now removed.

“Here we update *Schistosoma* and *Bulinus* species distributions in Zanzibar as a schistosomiasis surveillance resource, while also investigating specific associations between *Bulinus* and *Schistosoma* spp. on both Pemba and Unguja islands.”

Line 434. “negating” is the wrong word. Maybe “refuting”.

Corrected.

Line 452. Spelling of “conductivity”.

Corrected. Apologies for typo not picked up in spell check.

Line 456. Be consistent in use (or not) of am initial capital letter in “archipelago”.

Corrected. All capital A.

Lines 506-509. Use fewer words. “ the cost and time required to generate cox1 sequence data limited the number of the snails that could be identified by this means”. Or something similar.

Thanks for suggestion, sentence now reduced:

“The time and costs required to generate *cox*1 sequence data limited the number of snail intermediate hosts that could be identified by this means.”

Reviewer #3: I would like to thanks the authors for considering my comments and those of my colleagues. I found that the manuscript is much clearer and easier to follow as it is now presented.

Thanks to reviewer 3 for taking the time to check over the revision again.

**Further minor changes:**

Line 74: ‘Schistosomiasis’ changed to ‘Schistosome’

Line 120: ‘previously’ changed to ‘earlier’ to avoid repetition

Line 420: added ‘Most (n = 13 of 18) *S. haematobium cox*1 haplotypes’
